# Comparative evaluation of volume and helical four‐dimensional computed tomography using canon system

**DOI:** 10.1002/acm2.70351

**Published:** 2025-11-14

**Authors:** Changhwan Kim, Eunho Lee, Jae Hong Yu, Hojae Kim, Seyjoon Park, Soorim Han, Tae Ho Kim, Min Cheol Han, Chae‐Seon Hong, Jin Sung Kim

**Affiliations:** ^1^ Department of Radiation Oncology, Yonsei Cancer Center Heavy Ion Therapy Research Institute Yonsei University College of Medicine Seoul South Korea; ^2^ Department of Radiation Oncology, Yonsei Cancer Center Heavy Ion Therapy Research Institute Seoul South Korea; ^3^ Oncosoft Inc Seoul South Korea

**Keywords:** 4DCT, helical scan mode, respiratory‐gated radiotherapy, volume scan mode

## Abstract

**Background:**

Four‐dimensional computed tomography (4DCT) is an advanced imaging technique designed to capture anatomical motion over time, enabling more accurate evaluation of tumor and organ motion owing to respiration, while minimizing motion‐related artifacts. 4DCT employs two primary scanning modes—volume (cine) mode and helical mode—each with distinct clinical implications. A comprehensive evaluation of both approaches is therefore essential prior to clinical implementation.

**Purpose:**

This study aimed to compare volume and helical 4DCT scan modes in terms of image quality, dimensional accuracy, and positional accuracy for respiratory‐gated radiotherapy.

**Methods:**

4DCT imaging was conducted using a Canon Aquilion ONE Prism CT scanner integrated with an Anzai respiratory gating system. The two scan modes were assessed under identical imaging parameters, with differences arising only from their respective acquisition protocols. Phantom experiments were conducted using a motion phantom to simulate normal, rapid, and irregular respiratory patterns. Image quality, dimensional accuracy, and positional accuracy were quantitatively evaluated. Additionally, a retrospective analysis was conducted on clinical 4DCT datasets (*n* = 10), including lung, liver, and pancreatic cancers.

**Results:**

Volume mode demonstrated superior dose efficiency and dimensional consistency, preserving stable object volumes across respiratory phases, particularly under rapid motion conditions. Helical mode achieved higher positional accuracy and improved temporal resolution for motion tracking, but with a greater imaging dose. These findings were consistent with the clinical datasets, wherein volume mode reduced dimensional errors and helical mode provided more accurate motion representation.

**Conclusions:**

Both volume and helical 4DCT modes offer distinct advantages depending on clinical objectives. Volume mode is better suited for applications requiring high spatial accuracy and reduced radiation exposure, while helical mode is preferable for precise tracking of respiratory motion owing to its superior temporal resolution. Careful selection of the scan mode and system settings is essential for optimizing respiratory‐gated radiotherapy planning.

## 1 INTRODUCTION

Four‐dimensional computed tomography (4DCT) is an advanced imaging technique that extends conventional three‐dimensional CT (3DCT) by capturing anatomical motion over time.^1^, ^2^, ^3^, ^4^, ^5^, ^6^, ^7^, ^8^, ^9^ This is achieved by repeatedly scanning over a defined time interval, during which the acquired images are temporally synchronized with respiratory signals recorded using an external surrogate or other respiratory monitoring systems. By accounting for respiratory motion, 4DCT enables more accurate assessment of tumor and organ motion owing to respiration while reducing motion‐induced artifacts. Consequently, 4DCT is widely adopted in radiation therapy to reduce the adverse impact of respiratory motion on treatment accuracy. Its use is particularly critical for thoracic and abdominal tumors, where respiration‐induced motion can be substantial.^10^, ^11^, ^12^, ^13^, ^14^, ^15^ Image acquisition in 4DCT typically involves dividing a single respiratory cycle into multiple phase bins—commonly ten—and sorting the acquired images accordingly. By delineating the tumor and surrounding organs in each respiratory phase, 4DCT facilitates the visualization of their motion throughout the respiratory cycle. Such detailed motion characterization supports more precise delineation of the treatment volume while minimizing radiation exposure to surrounding healthy tissues.

Two principal scan modes are used in 4DCT: volume (cine) mode and helical mode.^16^, ^17^, ^18^, ^19^, ^20^ In the volume mode, images are acquired during stationary couch positions, allowing a large dataset to be collected at each location. To ensure complete coverage of all respiratory phases, the acquisition time per couch position typically exceeds one respiratory cycle. The couch is then moved to the next position, and the process is repeated. A key advantage of volume mode is its ability to maintain consistent spatial resolution, as image acquisition occurs without couch movement. However, inconsistencies in respiratory patterns between successive couch positions can cause discontinuities at the boundaries of the detector's field of view (FOV), manifesting as stair‐step artifacts that are often unavoidable.

The helical mode acquires images while the couch moves continuously at a constant speed, with the velocity determined by the pitch value. Pitch is defined as the ratio of the couch travel per gantry rotation to the x‐ray beam collimation width and can be adjusted to control the temporal resolution. To accurately capture motion, each anatomical slice must be imaged over at least one complete respiratory cycle. Therefore, the ratio of gantry rotation time to pitch must be greater than or equal to the duration of the respiratory cycle. Unlike the step‐and‐shoot acquisition used in volume mode, which requires the couch to stop at fixed intervals, helical mode enables uninterrupted scanning over extended anatomical regions. Furthermore, volume mode may lead to variations in image acquisition timing between couch positions, resulting in insufficient data coverage during specific phases of the respiratory cycle. In contrast, helical mode provides more consistent and uniform data acquisition throughout the respiratory cycle. However, because of its continuous scanning nature, images from adjacent slices may become misaligned, necessitating interpolation and correction during reconstruction—a limitation that may adversely affect spatial resolution.

The volume and helical 4DCT scan modes offer distinct advantages and limitations, with important implications for clinical practice. In addition, individual manufacturers implement proprietary hardware and software correction algorithms to compensate for these limitations, resulting in variability across systems. As such, a thorough understanding and comparative evaluation of these characteristics and techniques are essential prior to clinical adoption. Selecting the appropriate scan mode based on the patient's respiratory pattern and treatment site is critical for achieving optimal image quality and accurate radiation therapy planning. This study presents a practical comparative analysis of volume and helical 4DCT modes implemented in the Canon Aquilion ONE Prism system used at our institution, utilizing both phantom experiments and patient datasets. Although previous studies^1^, ^16^, ^20^ have investigated the principles and differences between these scan modes, most have focused on theoretical considerations or feasibility studies under highly controlled conditions. To date, comprehensive evaluations incorporating clinically relevant metrics, such as image quality, dimensional accuracy and positional accuracy, under realistic respiratory scenarios remain limited. This study addresses this gap by quantitatively assessing the performance of both scan modes across a range of respiratory patterns, thereby providing empirical evidence to guide scan mode selection and support clinical decision‐making in radiation therapy planning.

## 2 METHODS

### 2.1 Experimental conditions

All 4DCT data in this study were acquired using the Aquilion ONE Prism Edition (Canon Medical Systems, Otawara, Japan) system, a CT scanner equipped with 320 detector rows that provided a maximum *Z*‐axis coverage of 16 cm. This configuration allows for wide‐range imaging in a single gantry rotation without requiring table movement. Respiratory signal acquisition and synchronization with CT scanning were achieved using the AZ‐733VI respiratory gating system with a laser sensor (Anzai Medical, Tokyo, Japan). Based on the respiratory signals obtained from the external surrogate system, the 4DCT images were sorted into 10 respiratory phases.

To ensure a consistent comparison between the two scan modes, all imaging conditions were kept identical except for the mode‐specific acquisition parameters. The common CT scan and reconstruction parameters, along with the external surrogate system settings, are summarized in Table 1.

**TABLE 1:** Acquisition and reconstruction parameters used for 4DCT imaging in this study.

System	Parameter type	Parameter	Value
CT	Scan	Tube voltage	120 kVp
		Tube current	100 mA
		Rotation time	0.5 s
	Reconstruction	Reconstruction algorithm	AiCE
		Slice thickness	1 mm
		FOV	500 mm
		Matrix size	512 × 512 pixels
		Pixel Spacing	0.976 mm/pixel
Respiratory gating	Gate (Trigger)	Peak (In‐Peak)^*^	

* Range of Gate output level was disabled.

Abbreviation: AiCE, advanced intelligent clear‐IQ engine.

Certain scan parameters were configured differently to accommodate the inherent characteristics of each mode. In the volume mode, the total collimation width was set to 140 mm, whereas in the helical mode, the collimation width and pitch were set to 80 mm and 0.073, respectively. These settings were selected according to vendor recommendations. The resulting 4DCT images obtained from the volume and helical scan modes were then compared under these conditions.

### 2.2 Experimental study with phantom

**FIGURE 1:**
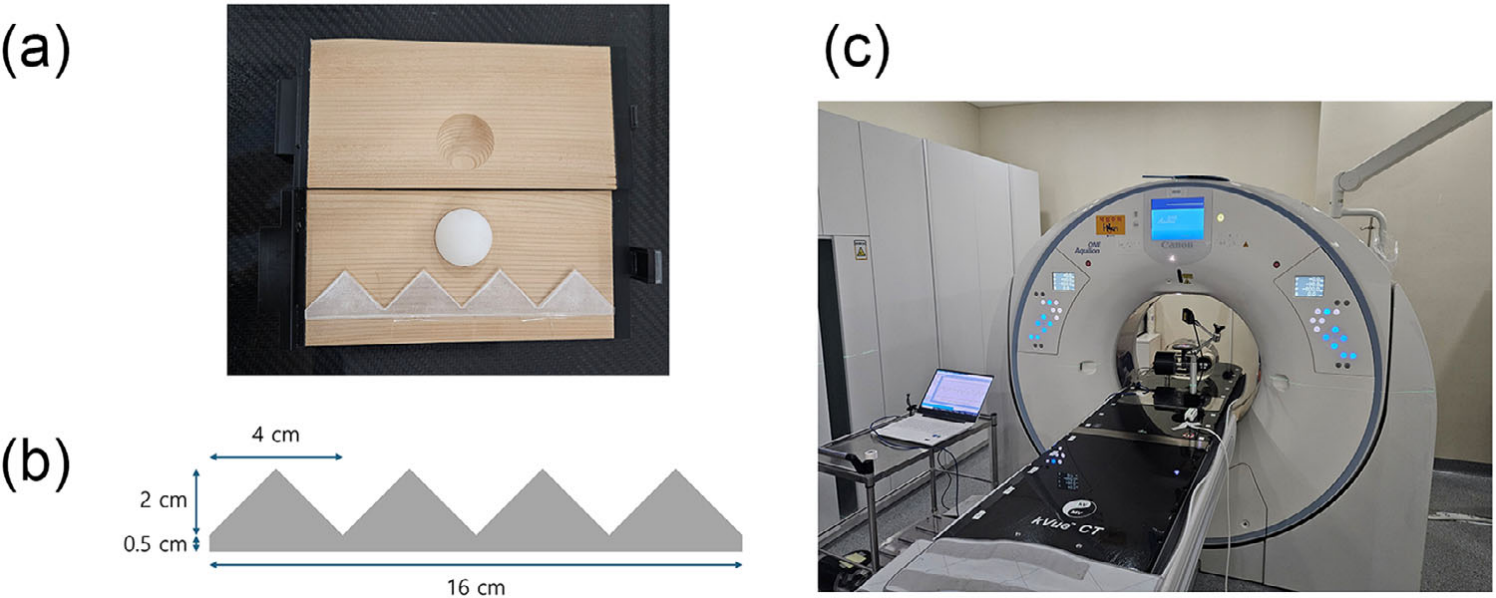
Experimental setup for the phantom study. (a) Cross‐sectional view of the insert module containing the custom‐made structure within the Quasar motion phantom. (b) Design of the sawtooth‐shaped plastic structure used for qualitative assessment of motion artifacts. (c) CT system and phantom configuration used for 4DCT imaging.

An experimental study was first conducted using a motion phantom to quantitatively compare the 4DCT images acquired in the volume and helical scan modes. The Quasar motion phantom (IBA Quasar, London, Canada) was employed for this purpose. The phantom's insert module includes a manufacturer‐provided spherical target with a diameter of 3 cm, designed to simulate a moving tumor. Additionally, a custom‐made sawtooth‐shaped structure composed of 1.2 mm thick polypropylene was incorporated to enhance motion artifact visualization and enable qualitative evaluation, as shown in Figure 1a,b. For the experiment, a sinusoidal motion pattern with an amplitude of 1 cm and a period of 4 s (maximum velocity: 1.57 cm/s) was used to simulate a typical respiratory cycle. The phantom was operated under this motion input, and 4DCT scans were acquired separately using both scan modes. The resulting images were evaluated for image quality relative to imaging dose, as well as for dimensional and positional accuracy in each mode. The overall phantom setup and CT scanning environment are shown in Figure 1c.

**FIGURE 2:**
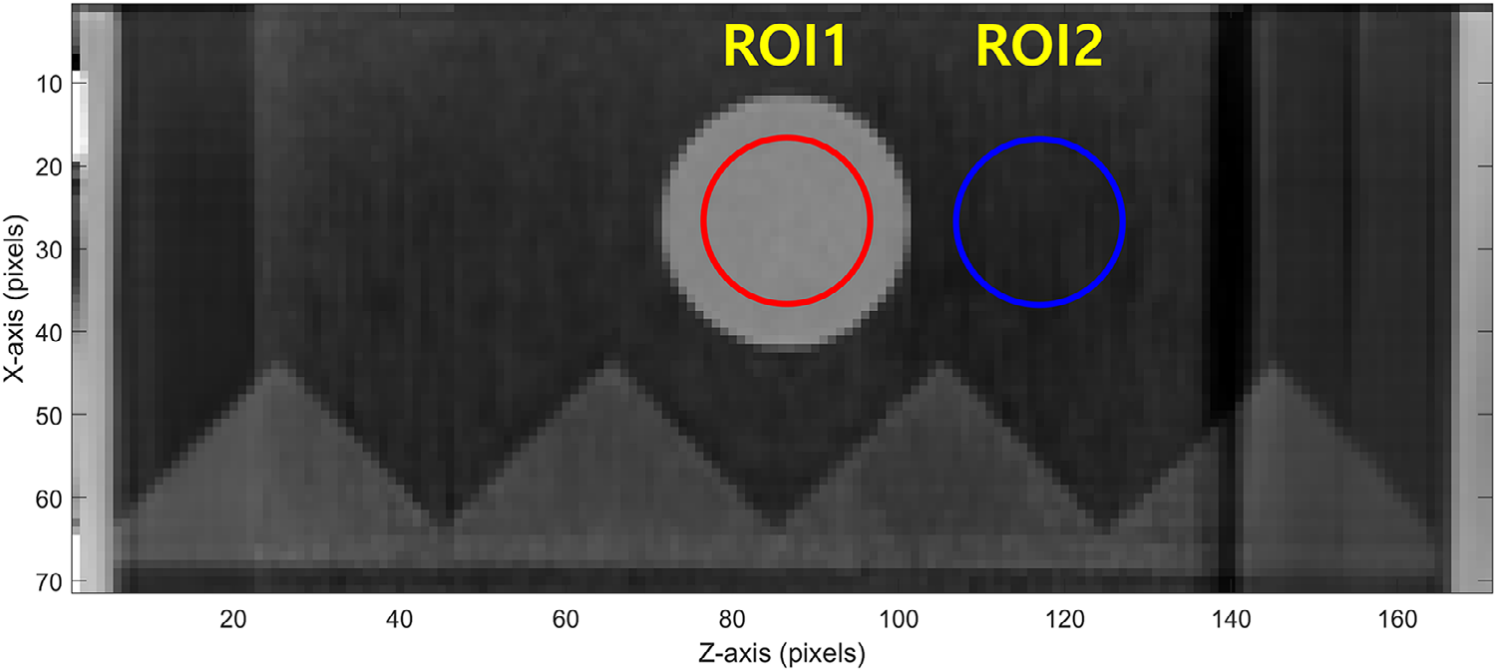
Placement of regions of interest (ROIs) for CNR calculation across respiratory phases in 4DCT images.

For image quality analysis, the contrast‐to‐noise ratio (CNR) was quantitatively evaluated with respect to the delivered dose to identify which scan mode provided more dose‐efficient 4DCT imaging. As depicted in Figure 2, two spherical regions of interest (ROIs), each with a diameter of 20 pixels, were defined: one centered within the target and the other positioned 30 pixels away from it. For each respiratory phase, Hounsfield Unit (HU) values were extracted from both ROIs, and their mean and standard deviations were computed. The CNR was then calculated using Equation (1) based on these values:

(1) $$CNR=\frac{M_{ROI1}-M_{ROI2}}{\sqrt{\frac{SD_{ROI1}^{2}+SD_{ROI2}^{2}}{2}}},$$

where M represents the mean and SD represents the standard deviation of the ROI.

(2) $$\mathrm{Dose}-\mathrm{normalized}\;\mathrm{CNR}\;=\frac{CNR}{CTDI_{vol}}$$

The volume CT dose index ($CTDI_{vol}$), used as an indicator of imaging dose, was extracted from the CT console. Using these values, the dose‐normalized CNR was calculated using Equation (2) to evaluate the dose efficiency of image quality. To evaluate dimensional accuracy, the volume of the spherical target was measured across all respiratory phases of the 4DCT scans, enabling assessment of motion‐induced distortions in spatial representation. In this context, dimensional accuracy refers to the degree to which the physical dimensions of the object were preserved in the reconstructed images. A qualitative evaluation was performed using the previously described sawtooth‐shaped plastic structure to further evaluate motion artifacts. For positional accuracy, the target's location in each phase was determined by calculating the center of mass of the spherical target. These measurements were compared against the known motion input to evaluate how accurately each scan mode reproduced the time‐resolved trajectory of the moving target throughout the respiratory cycle. The ground truth for positional accuracy was defined by the sinusoidal motion applied to the phantom. To reduce variability from manual contouring and ensure consistency in the assessment of both dimensional and positional accuracy, a threshold‐based segmentation approach was employed using a fixed HU criterion of –200 HU. This threshold was selected based on its ability to produce a contour that is most consistent with the known physical size (3 cm diameter) of the sphere, as verified using 3DCT images acquired separately in both volume and helical modes.

Moreover, the robustness of each 4DCT scan mode was evaluated under three respiratory patterns: normal, rapid, and irregular breathing. To simulate rapid motion, the amplitude of the reference signal was increased to 2 cm, corresponding to a maximum velocity of 3.14 cm/s. For the irregular motion scenario, random variations in amplitude and period were introduced into the normal respiratory pattern, resulting in a breathing signal with 15% irregularity.^21^, ^22^ For both rapid and irregular motion conditions, the same three evaluation metrics—image quality, dimensional accuracy, and positional accuracy—were applied to investigate the impact of motion variability on each scan mode.

### 2.3 Experimental study with patient data

In addition to the phantom experiments, a comparative analysis of 4DCT images acquired using volume and helical scan modes was conducted using clinical patient data. At our institution, patients underwent pre‐scan respiratory training to promote stable and consistent breathing patterns during both CT acquisition and subsequent respiratory‐gated radiotherapy. Following training, 4DCT imaging was performed using both scan modes, and the most appropriate dataset was selected for treatment planning. By performing consecutive volume and helical scans from the same patient within the established clinical workflow, including standardized respiratory training, 4DCT data were obtained under comparable respiratory conditions.

**FIGURE 3:**
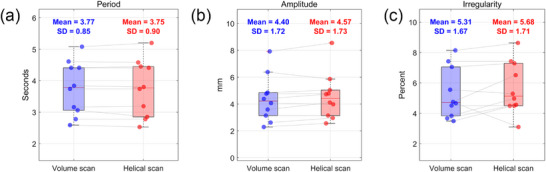
Comparison of respiratory metrics between volume and helical scan modes in selected patients: (a) Respiratory period, (b) amplitude, and (c) irregularity. Each data point represents a patient; lines connect paired values from the same patient. Mean and standard deviation (SD) are indicated for each group (*n* = 10). Statistical equivalence was confirmed using the two one‐sided test (TOST) method (equivalence margin: 5% of the mean).

This study retrospectively analyzed 4DCT datasets between March and April 2025 from 10 patients who were diagnosed with lung, liver, or pancreatic cancer and underwent 4DCT scanning for respiratory‐gated radiotherapy. Data collection was approved by the institutional review board (IRB) of the affiliated institution, and all patient information was anonymized prior to analysis. Table 2 summarizes the disease type and key respiratory parameters for each patient, including respiratory period, amplitude, and irregularity, derived from the external surrogate signal recorded using the AZ‐733VI system. As shown in Figure 3, respiratory characteristics were consistent across the two scan modes. To further confirm the comparability of the datasets, an equivalence test was performed using the two one‐sided test (TOST) method. This method not only evaluates the absence of significant differences between groups but also evaluates whether observed differences fall within a predefined equivalence margin, thereby establishing practical equivalence. In this study, the equivalence margin was conservatively defined as 5% of the mean value. The test results confirmed statistical equivalence between the respiratory patterns observed in volume and helical scans. These findings indicate that the effect of scan order on respiratory behavior was negligible, supporting the validity of the comparative analysis under comparable respiratory conditions.

**TABLE 2:** Summary of disease sites and respiratory characteristics for the patients included in this study.

			Volume scan			Helical scan		
Patient No.	Disease site	Evaluation ROI	Period (sec)	Amplitude (mm)	Irregularity (%)	Period (sec)	Amplitude (mm)	Irregularity (%)
#1	Liver	Liver	2.59	4.77	8.14	2.53	4.78	8.63
#2	Liver	Liver	4.41	7.92	5.55	4.45	8.55	5.29
#3	Lung	Target	3.16	2.62	7.05	3.19	2.96	7.29
#4	Lung	Target	3.06	4.07	4.66	2.85	3.98	3.1
#5	Lung	Target	4.41	3.14	7.42	4.41	3.14	7.42
#6	Lung	Target	3.84	4.38	4.75	3.8	4.73	6.51
#7	Pancreas	Stomach	2.78	3.59	3.83	2.78	4.11	4.97
#8	Pancreas	Gallbladder	4.61	2.29	3.49	4.58	2.56	4.56
#9	Pancreas	Stomach	3.74	6.38	4.51	3.74	5.86	4.5
#10	Pancreas	Gallbladder	5.08	4.85	3.67	5.2	5.04	4.49

Total			3.77	4.40	5.31	3.75	4.57	5.68

Abbreviation: ROI, regions of interest.

A quantitative comparison was conducted using the same methodology as applied in the phantom study. However, image quality assessment was excluded from the patient analysis owing to the inherent variability in patient anatomy and the absence of a ground truth reference, which made direct comparisons unreliable. Dimensional and positional accuracy were evaluated based on the contours of the target or adjacent normal organs identified in the 4DCT images. When the tumor was clearly identifiable, manual segmentation was performed to delineate the target. In cases with limited tumor visibility, OncoStudio version 2.0 (OncoSoft, Seoul, Republic of Korea), an auto‐segmentation software for normal organs, was employed to minimize operator‐dependent variability and bias, thereby ensuring a more objective evaluation. The contours generated through auto‐segmentation were visually double‐checked by the research team to confirm consistency across all respiratory phases. In cases where segmentation inaccuracies were identified—typically in regions with poorly defined boundaries—manual corrections were applied. For each patient, one organ adjacent to the tumor was selected from the auto‐segmented structures for quantitative evaluation.

For dimensional accuracy, the volume of the selected organ or target was measured across all respiratory phases. Since organs are not rigid and their volumes can vary by phase according to respiration, a composite metric, integrated HU volume, was calculated by multiplying the segmented volume by the corresponding mean HU. This approach provides a more robust indicator of dimensional consistency by incorporating tissue density variations. As both volume and integrated HU values vary across patients, direct comparisons of absolute values were not feasible. Instead, the mean and standard deviations of each metric were calculated across phases, and the coefficient of variation (CoV) was derived by dividing the standard deviation by the mean to enable normalization.

To evaluate positional accuracy, the center‐of‐mass coordinates of the selected structure were calculated for each respiratory phase to map its motion trajectory. As ground truth positional data are not available for patient studies, an indirect evaluation was performed by calculating the correlation between the structure's phase‐specific motion and the external respiratory surrogate signal. This correlation served as a measure of temporal precision in tracking respiratory motion.

## 3 RESULTS

### 3.1 Phantom data results

Phantom scans and data acquisition were performed for each 4DCT scan mode and respiratory pattern following the methodology described in the Methods section. To ensure result reliability, each scan was repeated three times under identical conditions. As the repeated measurements exhibited minimal variation, only one representative dataset was selected for analysis here to maintain clarity and conciseness. The complete set of results from all three trials is provided in the Supporting Information. The corresponding quantitative values are summarized in Table 3. Minimal variation was observed in CNR values across different respiratory patterns within the same scan mode. However, CT images acquired using the helical mode exhibited approximately 9% higher CNR compared to those obtained with the volume mode. Despite this, the imaging dose for the helical mode (33.8 mGy) was higher than that for the volume mode (27 mGy), resulting in a dose‐normalized CNR that was approximately 15% higher in the volume mode. This indicates superior dose efficiency for image quality in the volume mode.

**TABLE 3:** Quantitative comparison of image quality, dose efficiency, and scan time for the volume and helical scan modes using representative phantom data. Metrics include CNR, $CTDI_{vol}$, and dose‐normalized CNR.

Scan mode	Respiration type	$M_{\mathrm{ROI}1}$	$SD_{\mathrm{ROI}1}$	$M_{\mathrm{ROI}2}$	$SD_{\mathrm{ROI}2}$	$CNR_{\mathrm{ind}}$	$CNR_{\mathrm{avg}}$	$CTDI_{\mathrm{vol}}$ [mGy]	Dose‐normalized CNR	Scan time (s)
Volume	Normal	−40.95	11.61	−690.71	61.60	14.66	14.61	27.00	0.54	15.6
	Rapid	−41.30	11.41	−689.78	62.14	14.52				
	Irregular	−40.38	11.57	−691.34	61.77	14.65				
Helical	Normal	−44.48	14.16	−684.75	55.02	15.94	15.88	33.80	0.47	26.6
	Rapid	−44.37	14.28	−684.05	54.86	15.96				
	Irregular	−44.35	14.38	−685.41	55.81	15.73				

Abbreviations–ROI: regions of interest; M: mean; SD: standard deviation; $CNR_{\mathrm{ind}}$: contrast‐to‐noise ratio (individual); $CNR_{\mathrm{avg}}$: contrast‐to‐noise ratio (averaged); $CTDI_{\mathrm{vol}}$: volume CT dose index.

**FIGURE 4:**
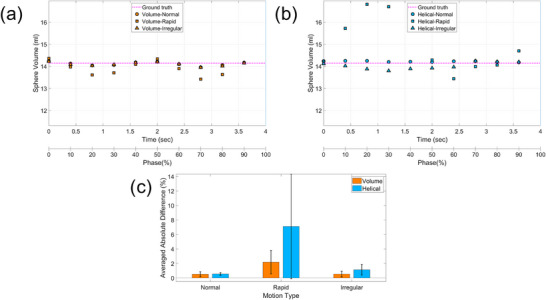
Dimensional accuracy in the phantom across different respiratory patterns and scan modes from a representative phantom dataset. (a) Phase‐specific volume variation in volume mode. (b) Phase‐specific volume variation in helical mode. (c) Averaged absolute volume difference from the ground truth for each respiratory pattern.

Dimensional accuracy was evaluated by analyzing the volume of the spherical target across respiratory phases, as shown in Figure 4. Both scan modes exhibited slight variations in measured volume across phases, primarily owing to motion artifacts. Under normal and irregular respiratory conditions, target volume remained relatively stable throughout the respiratory cycle, with negligible differences between the scan modes. However, during rapid respiration, large deviations from the reference volume were observed for both modes, with more pronounced errors in the helical scans. The largest discrepancies occurred within the 20%–30% phase interval, corresponding to the peak velocity of target motion. These discrepancies in the helical scans are likely attributable to temporal blurring or reconstruction inaccuracies associated with continuous gantry rotation.

**FIGURE 5:**
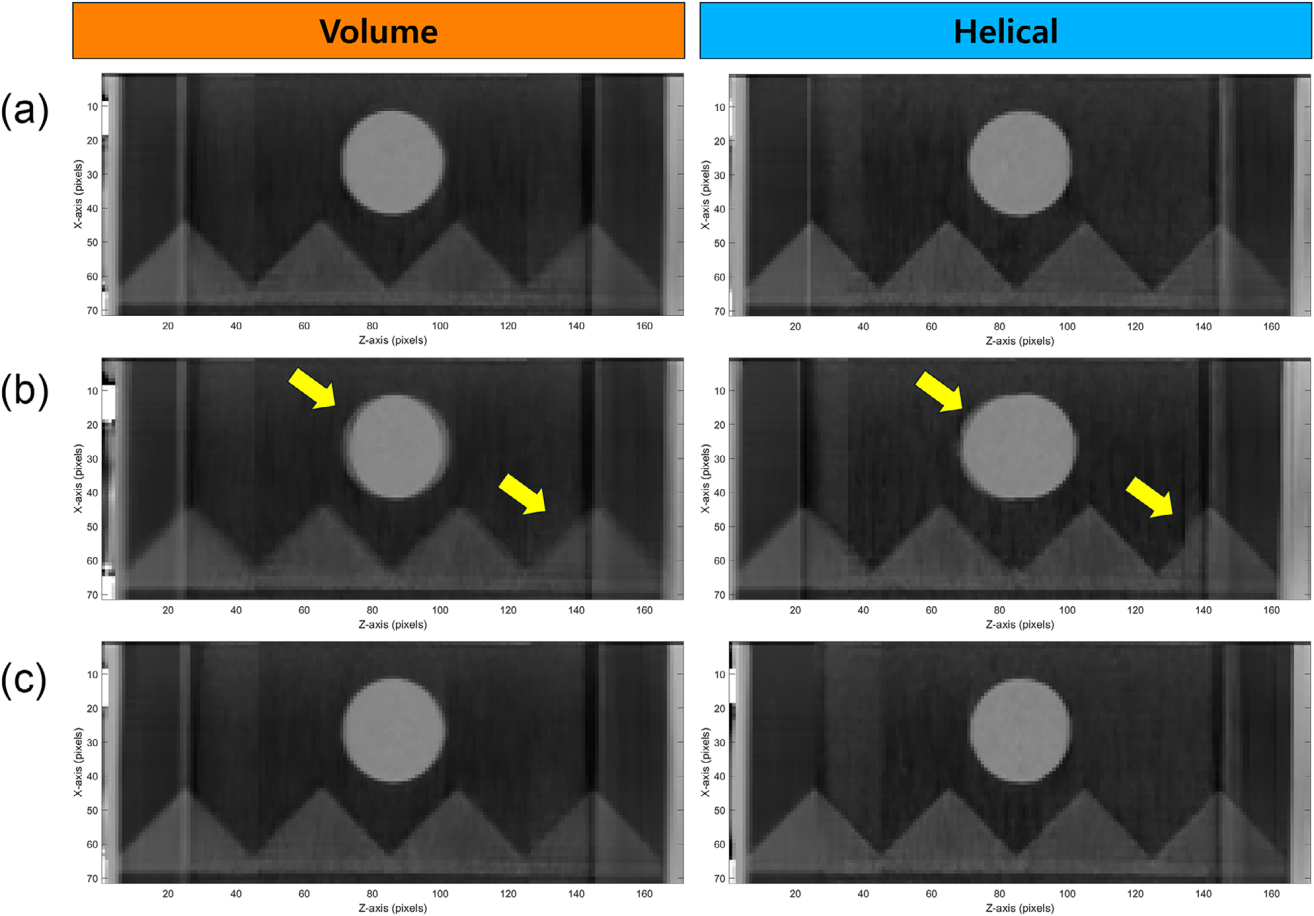
Coronal 4DCT images at the 20% respiratory phase acquired using volume (left column) and helical (right column) scan modes under different breathing patterns: (a) normal, (b) rapid, and (c) irregular respiration.

This observation is further supported by the coronal CT images at the 20% respiratory phase, shown in Figure 5. Under normal and irregular respiratory patterns, the spherical target maintained a relatively circular shape in both scan modes. However, under rapid respiration, noticeable geometric distortions were observed. In the volume mode, the target exhibited slight edge blurring, while in the helical mode, the target appeared notably elongated, resulting in an elliptical distortion, as indicated by the yellow arrows. A similar trend was observed in the qualitative assessment using the sawtooth‐shaped structure within the motion phantom. For both normal and irregular breathing patterns, no substantial differences were noted between volume and helical scans. However, the edges of the sawtooth pattern were slightly more blurred in volume mode compared to helical mode. Under rapid respiration, motion artifacts became more prominent in both scan modes. In the volume mode, edge blurring occurred uniformly across the structure, while in the helical mode, artifacts manifested not only as blurring but also as localized discontinuities along the sawtooth boundaries, indicating a non‐uniform distribution of motion‐induced distortions.

In summary, both scan modes demonstrated acceptable dimensional accuracy under standard respiratory conditions. However, the volume mode exhibited greater robustness in preserving geometric fidelity under extreme respiratory motion.

**FIGURE 6:**
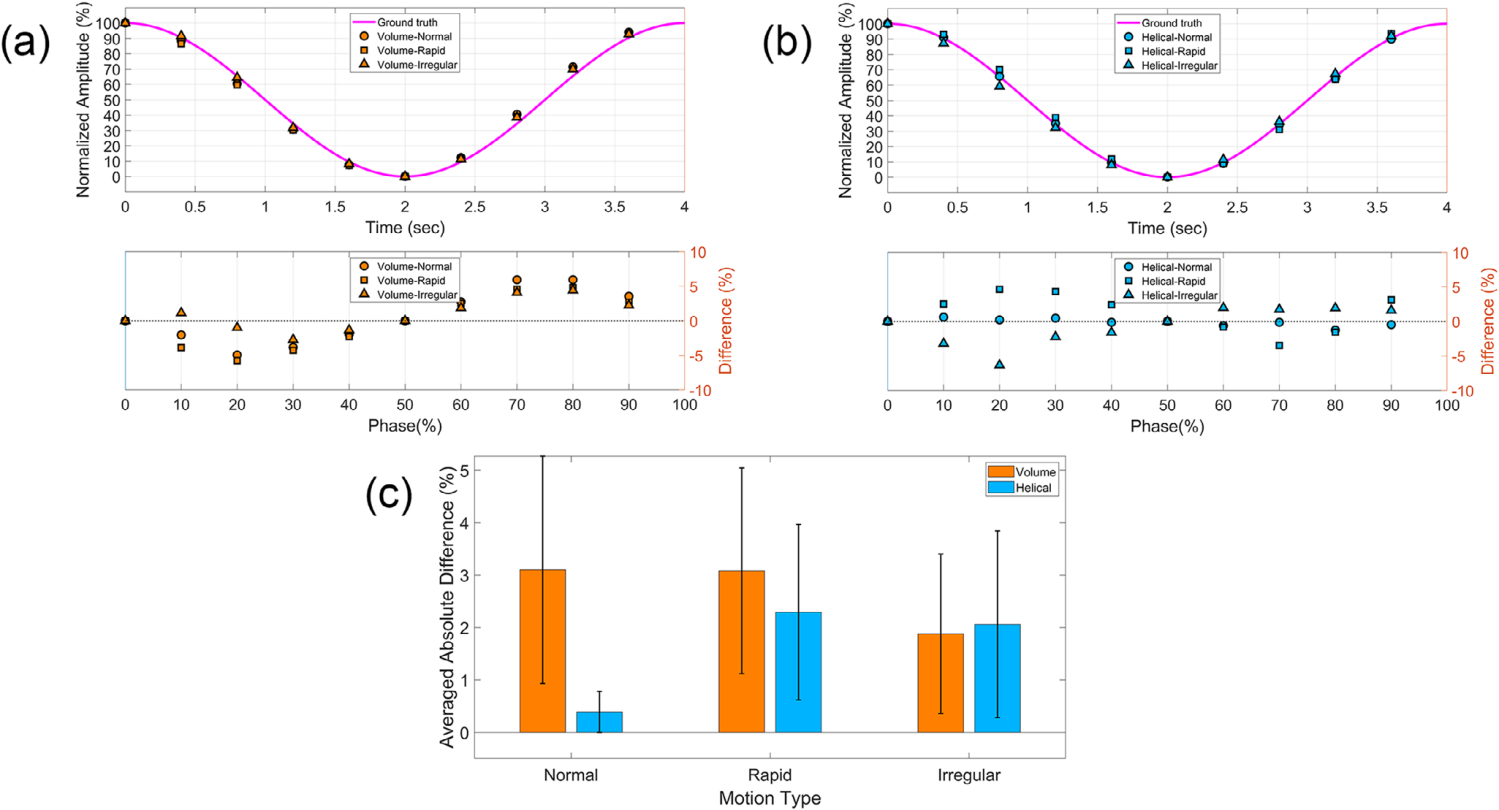
Positional accuracy across phases under different respiratory patterns and scan modes from a representative phantom dataset. (a) Target motion trajectory and positional errors in volume mode. (b) Target motion trajectory and positional errors in helical mode. (c) Averaged absolute positional error across phases for each respiratory pattern.

Positional accuracy was evaluated by comparing the center‐of‐mass position of the spherical target at each respiratory phase with the reference motion input of the phantom, as shown in Figure 6. In both scan modes, notable deviations were observed during phases associated with higher motion velocities. When evaluating the average absolute difference across all phases, the helical mode consistently exhibited smaller errors compared to the volume mode across all respiratory scenarios, suggesting better temporal fidelity in capturing time‐resolved target motion. This improved performance is likely attributable to the continuous data acquisition and finer temporal sampling inherent to the helical scanning approach.

### 3.2 Patient data results

**FIGURE 7:**
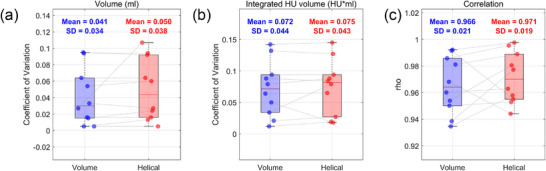
Dimensional and positional accuracy between volume and helical scan modes in patient data: (a) Coefficient of variation (CoV) of ROI volume. (b) CoV of integrated HU volume. (c) Correlation between internal 4DCT‐derived motion and external respiratory surrogate signal. Each data point represents an individual patient (*n* = 10).

Based on the phantom‐based analysis, a comparative evaluation was conducted using patient datasets to assess the dimensional and positional accuracy of 4DCT images acquired in volume and helical modes. Dimensional accuracy was assessed using the phase‐specific ROI volume and integrated HU volume, based on the ROI definitions provided in Table 2. Positional accuracy was evaluated by calculating the correlation between the center‐of‐mass positions of the ROI across respiratory phases and the external surrogate respiratory signal. The corresponding results are presented in Figure 7.

Although the differences were modest, both ROI volume and integrated HU volume exhibited lower CoV values in the volume mode compared with the helical mode. This indicates reduced phase‐to‐phase variability and improved consistency in volume mode, suggesting lower susceptibility to motion‐induced geometric errors. For positional accuracy, the correlation between the internal motion derived from 4DCT and the external surrogate signal was used as a measure of temporal accuracy. The average correlation coefficients were 0.966 for the volume mode and 0.970 for the helical mode, indicating that the latter provided marginally superior temporal accuracy in capturing respiratory‐induced motion.

In summary, the results from the patient data were consistent with those obtained from the phantom study: volume mode offered improved dimensional accuracy, while helical mode demonstrated better positional accuracy.

## 4 DISCUSSION

This study aimed to comprehensively compare and analyze the image quality, dimensional accuracy, and positional accuracy of volume and helical 4DCT scan modes using both phantom and patient datasets acquired with the Canon CT system. The primary objective was to provide practical guidance for clinical decision‐making in the context of respiratory‐gated radiotherapy planning. Through a systematic evaluation, the distinct characteristics and performance advantages of each scan mode were clearly identified, offering valuable insights into optimal 4DCT acquisition strategies in clinical practice.

The helical mode demonstrated a slightly higher CNR in phantom experiments; however, this came at the cost of a higher imaging dose. When comparing the two modes under matched imaging conditions, the volume mode exhibited superior dose efficiency, producing higher image quality per unit of radiation exposure. This finding supports its use in scenarios where minimizing patient dose is a priority. In terms of dimensional accuracy, both phantom and patient data confirmed that the volume mode better preserved spatial consistency across respiratory phases. On the other hand, the helical mode provided improved positional accuracy, capturing respiratory‐induced motion with greater temporal fidelity.

These findings highlight the importance of selecting the scan mode based on specific clinical objectives. For cases involving small target volumes or where preserving anatomical geometry is critical, the volume mode is preferable owing to its superior dimensional accuracy. In contrast, for clinical scenarios requiring high temporal resolution and precise motion tracking, such as moving tumors near critical structures, the helical mode may be more suitable. Additionally, the shorter scan time associated with volume mode can be advantageous for patients with irregular breathing patterns or limited ability to remain still for extended periods. However, the volume mode may be more susceptible to stair‐step artifacts in cases of irregular respiration. On the other hand, while the helical mode is less affected by respiratory irregularities owing to its continuous acquisition, its longer scan time may pose challenges for patients who cannot maintain consistent breathing. Coupled with the mode‐specific characteristics discussed in the Introduction, the findings of this study offer evidence‐based guidance for selecting the most appropriate 4DCT scan mode, thereby contributing to more precise and effective respiratory‐gated radiotherapy planning.

Although various previous studies have investigated different aspects of 4DCT imaging, their objectives and methodologies differ from those of the present work. Pan et al. conducted a comparative analysis of the helical and volume acquisition modes, focusing on data sufficiency conditions, slice sensitivity profiles, and acquisition time. However, their evaluation was limited to image acquisition and reconstruction processes, without a quantitative assessment of image quality degradation under irregular respiratory conditions. Their discussion of robustness against respiratory irregularity was largely conceptual, with the suggestion that re‐scanning may be feasible when using the volume mode. In an earlier study, Pan et al. evaluated patient data; however, their investigation was essentially a feasibility study aimed at confirming whether 4DCT can capture moving tumors and whether tumor contours were preserved. Matsuzaki et al. employed a CT system from the same manufacturer used in the present study and focused on optimizing 4DCT scanning protocols. However, their work did not incorporate respiratory motion simulation using a phantom and was therefore unable to fully reflect clinical conditions, although it did provide a comparative analysis of imaging dose. More recently, Yeo et al. also used the same CT system to assess the dimensional accuracy of 4DCT under different detector configurations (16 cm vs. 4 cm); however, their study did not compare scan modes nor did it include a quantitative evaluation of positional accuracy. In contrast, the present study provides a more comprehensive and clinically relevant evaluation of volume and helical 4DCT scan modes by quantitatively comparing image quality, dimensional accuracy, and positional accuracy using both phantom and clinical datasets. Furthermore, the robustness of each scan mode was evaluated under simulated rapid and irregular respiratory patterns. These contributions, particularly the integration of clinically representative motion conditions and the comprehensive quantitative analysis, represent the key contributions and novelty of this work, distinguishing it from previous studies.

**FIGURE 8:**
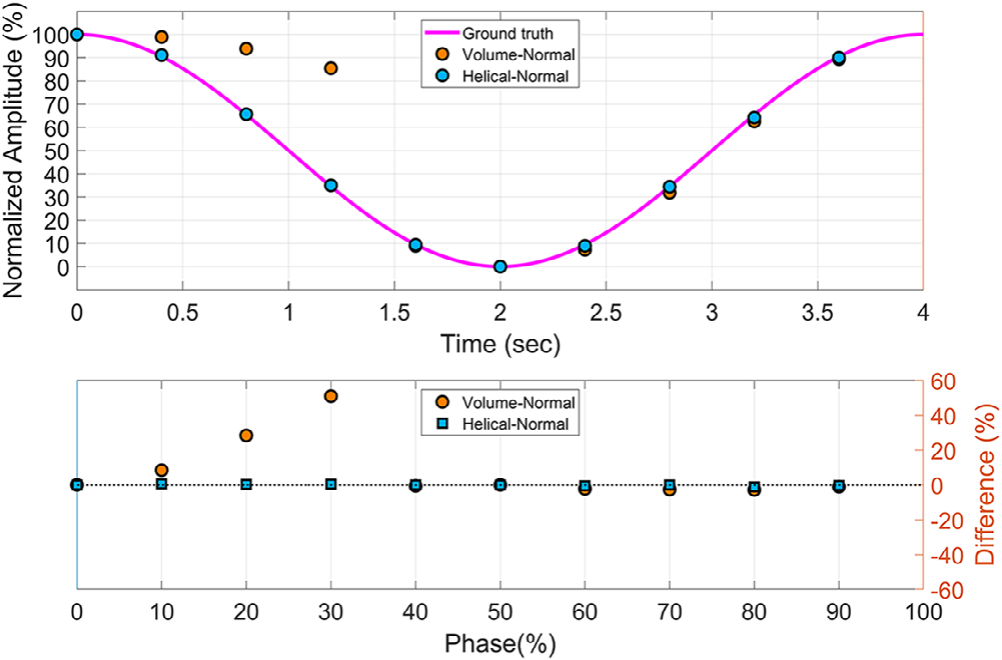
Positional accuracy across phases under the normal respiratory pattern using the point‐to‐point triggering configuration of the Anzai system. Comparison of target motion trajectory and positional error in volume and helical scan modes.

In addition to the aforementioned findings, an unexpected phenomenon was observed during the study: the positional accuracy of the Canon CT system was affected by the trigger configuration of the Anzai respiratory gating system. Specifically, when the x‐ray beam‐on trigger mode was switched from ‘Peak (In‐Peak)’ to ‘Point‐to‐Point,’ a phase shift of approximately 10%–20% was observed in the volume scan mode, resulting in a noticeable degradation of positional accuracy, as shown in Figure 8. Notably, this did not occur under the same conditions in the helical scan mode. This observation suggests that the trigger configuration of the Anzai system can introduce phase labeling error during retrospective 4DCT reconstruction, potentially owing to the way the Canon system's internal algorithms process respiratory signals. Since the helical mode involves continuous data acquisition and was unaffected by the trigger setting, the problem appears to be specific to the discrete acquisition nature of the volume mode. In the volume mode, respiratory information is sampled at specific time points. If the respiratory phase is inaccurately interpreted, it can result in either data omission or misalignment with the intended respiratory phase, leading to phase shift errors. Until this issue is addressed through a system‐level update, users should be aware of the potential for phase shift errors when using the “Point‐to‐Point” trigger mode in volume acquisitions. As a practical interim measure, configuring the Anzai system to use the “Peak (In‐Peak)” trigger setting is recommended to maintain positional accuracy in volume mode 4DCT scans.

Although this study identified several key findings, certain limitations must be acknowledged. First, all analyses were performed under a fixed set of scan and reconstruction parameters to ensure controlled comparisons between the volume and helical modes. Although this approach enhanced experimental consistency, it may not fully capture variations that can arise from changes in parameters such as pitch, collimation width, rotation time, or reconstruction algorithms. Alterations in these settings can influence image quality, the manifestation of motion artifacts, and overall reconstruction performance, potentially influencing the comparative results. Second, the study was conducted using a single CT system from one manufacturer. Although the mode‐dependent findings observed here are expected to hold across systems from other vendors in principle, differences in post‐processing and reconstruction algorithms may affect image characteristics. As such, caution should be exercised when generalizing the results to other systems. Third, a notable strength of this study lies in the use of paired patient data, enabling direct intra‐patient comparison of the two scan modes. However, the small cohort size—limited to 10 patients with varying disease sites—may restrict the statistical power and generalizability of the conclusions. Larger‐scale studies are needed to further validate and expand upon these findings.

Moreover, the assessment of positional accuracy in the patient study was performed indirectly by correlating the center‐of‐mass motion derived from 4DCT with the external surrogate respiratory signal. Although a high correlation implies good temporal consistency, it does not necessarily indicate true positional accuracy, as it relies on the assumptions and limitations of the external surrogate system. This method, although the most practical in a clinical setting where ground truth is unavailable, may be affected by individual patient factors such as target location and the motion dynamics of surrounding organs. Therefore, the correlation should be interpreted as an indirect measure of temporal tracking fidelity rather than a definitive indicator of positional accuracy.

## 5 CONCLUSION

This study presented a comprehensive comparative analysis of volume and helical 4DCT scan modes using both phantom and patient datasets acquired with the Canon CT system. The results demonstrated that the volume mode provided superior dimensional accuracy and greater dose efficiency, making it well‐suited for clinical scenarios where spatial fidelity and radiation dose minimization are prioritized. In contrast, helical mode demonstrated better positional accuracy, providing enhanced temporal resolution for capturing respiratory motion with greater precision. These findings highlight the importance of selecting the 4DCT scan mode based on specific clinical objectives, particularly the balance between geometric accuracy and temporal tracking performance. The insights gained from this study provide a foundation for optimizing 4DCT acquisition strategies in respiratory‐gated radiotherapy, ultimately contributing to enhanced treatment accuracy and improved patient outcomes.

## AUTHOR CONTRIBUTIONS

*Study concepts and design*: Changhwan Kim, Soorim Han, Tae Ho Kim, Min Cheol Han, Chae‐Seon Hong, and Jin Sung Kim. *Literature research*: Changhwan Kim, Eunho Lee, Jae Hong Yu, Hojae Kim, and Seyjoon Park. *Experimental studies and data analysis*: Changhwan Kim, Eunho Lee, Jae Hong Yu, Hojae Kim, Min Cheol Han, and Chae‐Seon Hong. *Statistical analysis*: Changhwan Kim, Soorim Han, Tae Ho Kim, Min Cheol Han, Chae‐Seon Hong, and Jin Sung Kim. *Manuscript preparation and editing*: Changhwan Kim, Min Cheol Han, and Chae‐Seon Hong. All authors read and approved the final manuscript.

## CONFLICT OF INTEREST STATEMENT

The authors declare that they have no competing interests.

## Ethic Statement

This study was approved by the institutional review board (IRB, approval number: 4‐2025‐0222) of Yonsei University Hospital. All data were fully anonymized before the investigators accessed them. The requirement for written informed consent was waived by the IRB because of the retrospective nature of the study.

## Supporting information



Supporting information
