# Supplementary material for: Comparative evaluation of volume and helical four‐dimensional computed tomography using canon system
Source: J Appl Clin Med Phys. 2025 Nov 14;26(11):e70351. doi: 10.1002/acm2.70351 (PMC12618175; doi:10.1002/acm2.70351)
Supplement: Supplementary file 1 — Supporting information [file ACM2-26-e70351-s001.docx]

Table S1. Quantitative comparison of image quality, dose efficiency and scan time between volume and helical scan modes for all three phantom measurement sets, including CNR, ${CTDI}_{vol}$, and dose-normalized CNR, with the second set (#2) designated as the representative measurement.

| Scan mode | Respiration Type | # | $M_{ROI1}$ | ${SD}_{ROI1}$ | $M_{ROI1}$ | ${SD}_{ROI2}$ | ${CNR}_{ind}$ | ${CNR}_{avg}$ | ${CTDI}_{vol}$ [mGy] | $\frac{{CNR}_{ind}}{{CTDI}_{vol}}$ | Dose-normalized CNR | | | Scan time (sec) |
| --- | --- | --- | --- | --- | --- | --- | --- | --- | --- | --- | --- | --- | --- | --- |
|  |  |  |  |  |  |  |  |  |  |  | Mean | SD | 95% CI |  |
| Volume | Normal | 1 | -40.939 | 11.576 | -690.616 | 61.528 | 14.675 | 14.604 | 27 | 0.544 | 0.541 | 0.003 | [0.539, 0.543] | 15.6 |
|  |  | 2 | –40.949 | 11.613 | -690.710 | 61.597 | 14.660 |  |  | 0.543 |  |  |  |  |
|  |  | 3 | -41.036 | 11.622 | -690.714 | 61.796 | 14.612 |  |  | 0.541 |  |  |  |  |
|  | Rapid | 1 | -41.377 | 11.440 | -689.928 | 62.202 | 14.502 |  |  | 0.537 |  |  |  |  |
|  |  | 2 | –41.297 | 11.410 | -689.780 | 62.143 | 14.515 |  |  | 0.538 |  |  |  |  |
|  |  | 3 | -41.236 | 11.362 | -689.523 | 62.033 | 14.538 |  |  | 0.538 |  |  |  |  |
|  | Irregular | 1 | -40.327 | 11.533 | -691.314 | 61.574 | 14.696 |  |  | 0.544 |  |  |  |  |
|  |  | 2 | –40.384 | 11.568 | -691.337 | 61.770 | 14.649 |  |  | 0.543 |  |  |  |  |
|  |  | 3 | -40.550 | 11.580 | -691.292 | 61.999 | 14.591 |  |  | 0.540 |  |  |  |  |
| Helical | Normal | 1 | -44.619 | 14.214 | -684.823 | 55.557 | 15.788 | 15.869 | 33.8 | 0.467 | 0.469 | 0.004 | [0.466, 0.473] | 26.6 |
|  |  | 2 | –44.476 | 14.165 | -684.748 | 55.021 | 15.937 |  |  | 0.472 |  |  |  |  |
|  |  | 3 | -44.448 | 14.145 | -684.576 | 54.537 | 16.068 |  |  | 0.475 |  |  |  |  |
|  | Rapid | 1 | -44.664 | 14.359 | -684.284 | 55.083 | 15.891 |  |  | 0.470 |  |  |  |  |
|  |  | 2 | –44.369 | 14.284 | -684.052 | 54.860 | 15.958 |  |  | 0.472 |  |  |  |  |
|  |  | 3 | -44.280 | 14.263 | -683.759 | 54.699 | 15.999 |  |  | 0.473 |  |  |  |  |
|  | Irregular | 1 | -44.447 | 14.380 | -685.231 | 55.605 | 15.778 |  |  | 0.467 |  |  |  |  |
|  |  | 2 | –44.349 | 14.380 | -685.414 | 55.814 | 15.730 |  |  | 0.465 |  |  |  |  |
|  |  | 3 | -44.402 | 14.400 | -685.601 | 56.050 | 15.669 |  |  | 0.464 |  |  |  |  |

Abbreviations–ROI: regions of Interest; $M$: mean; $SD$: standard deviation; ${CNR}_{ind}$: contrast-to-noise ratio (individual); ${CNR}_{avg}$: contrast-to-noise ratio (averaged); ${CTDI}_{vol}$: volume CT dose index; CI: confidence interval.

Table S2. Quantitative analysis of the spherical target volume in the phantom to evaluate dimensional accuracy across different respiratory patterns and scan modes for all three phantom measurement sets, with the second set (#2) designated as the representative measurement.

| Scan mode | Respiration Type | # | Volume (ml) | | | | | | | | | | Abs. Diff (%) | |
| --- | --- | --- | --- | --- | --- | --- | --- | --- | --- | --- | --- | --- | --- | --- |
|  |  |  | Phase (%) | | | | | | | | | |  |  |
|  |  |  | 0 | 10 | 20 | 30 | $40$ | 50 | 60 | 70 | 80 | 90 | Mean | SD |
| Volume | Normal | 1 | 14.22 | 14.13 | 14.04 | 14.04 | 14.17 | 14.19 | 14.12 | 13.95 | 14.06 | 14.15 | 0.48 | 0.38 |
|  |  | Diff (%) | 0.58 | -0.08 | -0.70 | -0.71 | 0.22 | 0.39 | -0.17 | -1.33 | -0.58 | 0.09 |  |  |
|  |  | 2 | 14.24 | 14.13 | 14.04 | 14.06 | 14.18 | 14.21 | 14.10 | 13.96 | 14.06 | 14.17 | 0.52 | 0.33 |
|  |  | Diff (%) | 0.71 | -0.10 | -0.69 | -0.54 | 0.28 | 0.51 | -0.28 | -1.26 | -0.59 | 0.21 |  |  |
|  |  | 3 | 14.24 | 14.11 | 14.05 | 14.07 | 14.19 | 14.21 | 14.08 | 13.95 | 14.08 | 14.15 | 0.52 | 0.35 |
|  |  | Diff (%) | 0.74 | -0.19 | -0.64 | -0.47 | 0.37 | 0.49 | -0.43 | -1.36 | -0.44 | 0.08 |  |  |
|  | Rapid | 1 | 14.36 | 13.96 | 13.60 | 13.72 | 14.09 | 14.33 | 13.91 | 13.42 | 13.63 | 14.17 | 2.18 | 1.60 |
|  |  | Diff (%) | 1.53 | -1.30 | -3.81 | -2.98 | -0.37 | 1.34 | -1.61 | -5.06 | -3.61 | 0.19 |  |  |
|  |  | 2 | 14.36 | 13.97 | 13.61 | 13.71 | 14.09 | 14.34 | 13.90 | 13.42 | 13.63 | 14.15 | 2.18 | 1.62 |
|  |  | Diff (%) | 1.59 | -1.20 | -3.72 | -3.02 | -0.37 | 1.39 | -1.72 | -5.09 | -3.62 | 0.04 |  |  |
|  |  | 3 | 14.35 | 13.98 | 13.63 | 13.71 | 14.09 | 14.32 | 13.87 | 13.42 | 13.64 | 14.17 | 2.16 | 1.60 |
|  |  | Diff (%) | 1.51 | -1.11 | -3.63 | -3.06 | -0.34 | 1.24 | -1.88 | -5.08 | -3.52 | 0.20 |  |  |
|  | Irregular | 1 | 14.24 | 14.08 | 14.05 | 14.06 | 14.17 | 14.18 | 14.12 | 13.93 | 14.03 | 14.17 | 0.54 | 0.41 |
|  |  | Diff (%) | 0.69 | -0.39 | -0.60 | -0.57 | 0.21 | 0.31 | -0.12 | -1.50 | -0.81 | 0.18 |  |  |
|  |  | 2 | 14.22 | 14.08 | 14.03 | 14.08 | 14.17 | 14.20 | 14.10 | 13.95 | 14.01 | 14.15 | 0.54 | 0.37 |
|  |  | Diff (%) | 0.57 | -0.40 | -0.78 | -0.42 | 0.23 | 0.45 | -0.29 | -1.33 | -0.90 | 0.07 |  |  |
|  |  | 3 | 14.24 | 14.06 | 14.02 | 14.07 | 14.19 | 14.20 | 14.12 | 13.94 | 14.00 | 14.13 | 0.60 | 0.41 |
|  |  | Diff (%) | 0.70 | -0.55 | -0.81 | -0.50 | 0.33 | 0.42 | -0.14 | -1.44 | -0.99 | -0.06 |  |  |
| Helical | Normal | 1 | 14.22 | 14.27 | 14.26 | 14.20 | 14.20 | 14.21 | 14.23 | 14.23 | 14.20 | 14.17 | 0.55 | 0.21 |
|  |  | Diff (%) | 0.60 | 0.92 | 0.81 | 0.42 | 0.39 | 0.49 | 0.66 | 0.62 | 0.39 | 0.24 |  |  |
|  |  | 2 | 14.24 | 14.25 | 14.25 | 14.20 | 14.21 | 14.19 | 14.23 | 14.24 | 14.20 | 14.18 | 0.55 | 0.19 |
|  |  | Diff (%) | 0.73 | 0.79 | 0.79 | 0.40 | 0.51 | 0.36 | 0.62 | 0.68 | 0.39 | 0.27 |  |  |
|  |  | 3 | 14.22 | 14.24 | 14.23 | 14.18 | 14.20 | 14.19 | 14.20 | 14.26 | 14.22 | 14.18 | 0.51 | 0.18 |
|  |  | Diff (%) | 0.58 | 0.69 | 0.65 | 0.29 | 0.42 | 0.33 | 0.46 | 0.82 | 0.55 | 0.27 |  |  |
|  | Rapid | 1 | 14.20 | 15.71 | 16.83 | 16.69 | 15.67 | 14.30 | 13.44 | 13.97 | 14.05 | 14.68 | 7.11 | 7.18 |
|  |  | Diff (%) | 0.41 | 11.09 | 19.02 | 18.05 | 10.81 | 1.13 | -4.97 | -1.17 | -0.61 | 3.85 |  |  |
|  |  | 2 | 14.20 | 15.72 | 16.81 | 16.70 | 15.69 | 14.29 | 13.44 | 13.99 | 14.06 | 14.70 | 7.11 | 7.19 |
|  |  | Diff (%) | 0.41 | 11.15 | 18.88 | 18.09 | 10.94 | 1.03 | -4.93 | -1.08 | -0.58 | 3.99 |  |  |
|  |  | 3 | 14.18 | 15.74 | 16.83 | 16.70 | 15.67 | 14.27 | 13.44 | 14.00 | 14.03 | 14.68 | 7.10 | 7.25 |
|  |  | Diff (%) | 0.28 | 11.30 | 19.04 | 18.12 | 10.79 | 0.94 | -4.98 | -0.97 | -0.75 | 3.83 |  |  |
|  | Irregular | 1 | 14.10 | 14.03 | 13.89 | 13.81 | 13.89 | 13.92 | 13.96 | 14.25 | 14.19 | 14.21 | 1.13 | 0.70 |
|  |  | Diff (%) | -0.26 | -0.77 | -1.78 | -2.32 | -1.75 | -1.53 | -1.25 | 0.79 | 0.36 | 0.51 |  |  |
|  |  | 2 | 14.12 | 14.02 | 13.88 | 13.80 | 13.89 | 13.92 | 13.97 | 14.24 | 14.21 | 14.20 | 1.13 | 0.73 |
|  |  | Diff (%) | -0.15 | -0.82 | -1.86 | -2.37 | -1.73 | -1.54 | -1.18 | 0.70 | 0.47 | 0.44 |  |  |
|  |  | 3 | 14.10 | 14.02 | 13.88 | 13.82 | 13.87 | 13.94 | 13.99 | 14.24 | 14.20 | 14.20 | 1.11 | 0.70 |
|  |  | Diff (%) | -0.26 | -0.87 | -1.81 | -2.27 | -1.88 | -1.39 | -1.08 | 0.69 | 0.45 | 0.42 |  |  |

Note: The ground truth of the spherical target volume was 14.14 ml

Abbreviations–$SD$: standard deviation; $Abs. Diff$: Absolute difference.

Table S3. Quantitative analysis of positional accuracy across phases under different respiratory patterns and scan modes for all three phantom measurement sets, with the second set (#2) designated as the representative measurement.

|  | | | Normalized Amplitude (%) | | | | | | | | | |  | |
| --- | --- | --- | --- | --- | --- | --- | --- | --- | --- | --- | --- | --- | --- | --- |
|  |  |  | Phase (%) | | | | | | | | | |  |  |
|  |  |  | 0 | 10 | 20 | 30 | $40$ | 50 | 60 | 70 | 80 | 90 |  |  |
| Reference | | | 100.00 | 90.45 | 65.45 | 34.55 | 9.55 | 0.00 | 9.55 | 34.55 | 65.45 | 90.45 |  |  |
|  | | | | | | | | | | | | | | |
| Scan mode | Respiration Type | # | Normalized Amplitude (%) | | | | | | | | | | Abs. Diff (%) | |
|  |  |  | Phase (%) | | | | | | | | | |  |  |
|  |  |  | 0 | 10 | 20 | 30 | $40$ | 50 | 60 | 70 | 80 | 90 | Mean | SD |
| Volume | Normal | 1 | 100.00 | 88.16 | 60.29 | 30.70 | 7.64 | 0.00 | 12.08 | 40.33 | 71.37 | 94.06 | 3.10 | 2.15 |
|  |  | Diff (%) | 0.00 | -2.29 | -5.16 | -3.85 | -1.91 | 0.00 | 2.53 | 5.78 | 5.92 | 3.61 |  |  |
|  |  | 2 | 100.00 | 88.40 | 60.50 | 30.70 | 7.60 | 0.00 | 12.30 | 40.50 | 71.40 | 94.00 | 3.10 | 2.17 |
|  |  | Diff (%) | 0.00 | -2.05 | -4.95 | -3.85 | -1.95 | 0.00 | 2.75 | 5.95 | 5.95 | 3.55 |  |  |
|  |  | 3 | 100.00 | 88.58 | 60.58 | 30.56 | 7.41 | 0.00 | 12.08 | 40.26 | 71.57 | 94.01 | 3.08 | 2.16 |
|  |  | Diff (%) | 0.00 | -1.87 | -4.87 | -3.99 | -2.14 | 0.00 | 2.53 | 5.71 | 6.12 | 3.56 |  |  |
|  | Rapid | 1 | 100.00 | 86.77 | 59.68 | 30.31 | 7.39 | 0.00 | 12.08 | 38.93 | 70.30 | 92.93 | 3.01 | 1.95 |
|  |  | Diff (%) | 0.00 | -3.68 | -5.77 | -4.24 | -2.16 | 0.00 | 2.53 | 4.38 | 4.85 | 2.48 |  |  |
|  |  | 2 | 100.00 | 86.57 | 59.67 | 30.28 | 7.26 | 0.00 | 12.08 | 39.13 | 70.26 | 93.14 | 3.08 | 1.96 |
|  |  | Diff (%) | 0.00 | -3.88 | -5.78 | -4.27 | -2.29 | 0.00 | 2.53 | 4.58 | 4.81 | 2.69 |  |  |
|  |  | 3 | 100.00 | 86.37 | 59.49 | 30.03 | 7.43 | 0.00 | 11.97 | 39.13 | 70.39 | 93.22 | 3.14 | 2.04 |
|  |  | Diff (%) | 0.00 | -4.08 | -5.96 | -4.52 | -2.12 | 0.00 | 2.42 | 4.58 | 4.94 | 2.77 |  |  |
|  | Irregular | 1 | 100.00 | 91.81 | 64.43 | 31.91 | 8.38 | 0.00 | 11.34 | 38.68 | 69.82 | 92.66 | 1.87 | 1.51 |
|  |  | Diff (%) | 0.00 | 1.36 | -1.02 | -2.64 | -1.17 | 0.00 | 1.79 | 4.13 | 4.37 | 2.21 |  |  |
|  |  | 2 | 100.00 | 91.59 | 64.46 | 31.83 | 8.21 | 0.00 | 11.41 | 38.64 | 69.87 | 92.69 | 1.88 | 1.52 |
|  |  | Diff %) | 0.00 | 1.14 | -0.99 | -2.72 | -1.34 | 0.00 | 1.86 | 4.09 | 4.42 | 2.24 |  |  |
|  |  | 3 | 100.00 | 91.64 | 64.68 | 31.81 | 8.23 | 0.00 | 11.28 | 38.82 | 69.68 | 92.59 | 1.84 | 1.53 |
|  |  | Diff (%) | 0.00 | 1.19 | -0.77 | -2.74 | -1.32 | 0.00 | 1.73 | 4.27 | 4.23 | 2.14 |  |  |
| Helical | Normal | 1 | 100.00 | 91.02 | 65.60 | 34.90 | 9.38 | 0.00 | 8.74 | 34.52 | 64.33 | 89.93 | 0.37 | 0.38 |
|  |  | Diff (%) | 0.00 | 0.57 | 0.15 | 0.35 | -0.17 | 0.00 | -0.81 | -0.03 | -1.12 | -0.52 |  |  |
|  |  | 2 | 100.00 | 91.07 | 65.67 | 35.02 | 9.42 | 0.00 | 8.93 | 34.42 | 64.19 | 89.98 | 0.39 | 0.39 |
|  |  | Diff (%) | 0.00 | 0.62 | 0.22 | 0.47 | -0.13 | 0.00 | -0.62 | -0.13 | -1.26 | -0.47 |  |  |
|  |  | 3 | 100.00 | 91.02 | 65.49 | 34.81 | 9.30 | 0.00 | 8.93 | 34.29 | 63.98 | 89.73 | 0.42 | 0.45 |
|  |  | Diff (%) | 0.00 | 0.57 | 0.04 | 0.26 | -0.25 | 0.00 | -0.62 | -0.26 | -1.47 | -0.72 |  |  |
|  | Rapid | 1 | 100.00 | 92.82 | 69.99 | 38.70 | 12.16 | 0.00 | 8.69 | 30.96 | 63.85 | 93.34 | 2.26 | 1.62 |
|  |  | Diff (%) | 0.00 | 2.37 | 4.54 | 4.15 | 2.61 | 0.00 | -0.86 | -3.59 | -1.60 | 2.89 |  |  |
|  |  | 2 | 100.00 | 92.98 | 70.11 | 38.88 | 11.96 | 0.00 | 8.75 | 31.08 | 63.88 | 93.58 | 2.29 | 1.67 |
|  |  | Diff (%) | 0.00 | 2.53 | 4.66 | 4.33 | 2.41 | 0.00 | -0.80 | -3.47 | -1.57 | 3.13 |  |  |
|  |  | 3 | 100.00 | 92.80 | 69.95 | 38.93 | 12.18 | 0.00 | 8.76 | 30.86 | 63.72 | 93.81 | 2.34 | 1.68 |
|  |  | Diff (%) | 0.00 | 2.35 | 4.50 | 4.38 | 2.63 | 0.00 | -0.79 | -3.69 | -1.73 | 3.36 |  |  |
|  | Irregular | 1 | 100.00 | 87.39 | 59.21 | 32.38 | 7.81 | 0.00 | 11.72 | 36.24 | 67.43 | 92.25 | 2.09 | 1.74 |
|  |  | Diff (%) | 0.00 | -3.06 | -6.24 | -2.17 | -1.74 | 0.00 | 2.17 | 1.69 | 1.98 | 1.80 |  |  |
|  |  | 2 | 100.00 | 87.26 | 59.12 | 32.31 | 7.95 | 0.00 | 11.52 | 36.29 | 67.38 | 92.05 | 2.06 | 1.78 |
|  |  | Diff (%) | 0.00 | -3.19 | -6.33 | -2.24 | -1.60 | 0.00 | 1.97 | 1.74 | 1.93 | 1.60 |  |  |
|  |  | 3 | 100.00 | 87.01 | 59.22 | 32.34 | 8.09 | 0.00 | 11.70 | 36.20 | 67.59 | 92.10 | 2.09 | 1.78 |
|  |  | Diff (%) | 0.00 | -3.44 | -6.23 | -2.21 | -1.46 | 0.00 | 2.15 | 1.65 | 2.14 | 1.65 |  |  |

Abbreviations–$SD$: standard deviation; $Abs. Diff$: Absolute difference.
